# Role of duodenal iron transporters and hepcidin in patients with alcoholic liver disease

**DOI:** 10.1111/jcmm.12310

**Published:** 2014-06-03

**Authors:** Marketa Dostalikova-Cimburova, Kamila Balusikova, Karolina Kratka, Jitka Chmelikova, Vaclav Hejda, Jan Hnanicek, Jitka Neubauerova, Jana Vranova, Jan Kovar, Jiri Horak

**Affiliations:** a Department of Cell and Molecular Biology, Third Faculty of Medicine, Charles University PraguePrague, Czech Republic; b Department of Medicine I, Third Faculty of Medicine, Charles University PraguePrague, Czech Republic; c 1st Dept. of Medicine, Charles University in Prague, Medical School and Teaching Hospital in PilsenPilsen, Czech Republic; d Department of Medicine II, Third Faculty of Medicine, Charles University PraguePrague, Czech Republic; e Department of Medical Biophysics and Informatics, Third Faculty of Medicine, Charles University PraguePrague, Czech Republic

**Keywords:** DMT1, FPN1, DCYTB, HEPH, TFR1, HFE, hepcidin, alcoholic liver disease, iron, gene expression

## Abstract

Patients with alcoholic liver disease (ALD) often display disturbed iron indices. Hepcidin, a key regulator of iron metabolism, has been shown to be down-regulated by alcohol in cell lines and animal models. This down-regulation led to increased duodenal iron transport and absorption in animals. In this study, we investigated gene expression of duodenal iron transport molecules and hepcidin in three groups of patients with ALD (with anaemia, with iron overload and without iron overload) and controls. Expression of DMT1, FPN1, DCYTB, HEPH, HFE and TFR1 was measured in duodenal biopsies by using real-time PCR and Western blot. Serum hepcidin levels were measured by using ELISA. Serum hepcidin was decreased in patients with ALD. At the mRNA level, expressions of *DMT1*, *FPN1* and *TFR1* genes were significantly increased in ALD. This pattern was even more pronounced in the subgroups of patients without iron overload and with anaemia. Protein expression of FPN1 paralleled the increase at the mRNA level in the group of patients with ALD. Serum ferritin was negatively correlated with *DMT1* mRNA. The down-regulation of hepcidin expression leading to up-regulation of iron transporters expression in the duodenum seems to explain iron metabolism disturbances in ALD. Alcohol consumption very probably causes suppression of hepcidin expression in patients with ALD.

## Introduction

Iron metabolism disturbances are common findings in patients with chronic alcohol consumption. It ranges from anaemia to iron overload [1–3]. The pathogenesis of anaemia in alcoholic liver disease (ALD) is complex; it includes hypersplenism with splenic pooling and increased destruction of erythrocytes, blood loss because of gastrointestinal bleeding as a complication of alcoholic cirrhosis, which can lead to anaemia with iron deficiency [4–8]. Inadequate diet with nutrient deficits, in alcoholic patients, can result in anaemia with megaloblastic and sideroblastic features [9]. Additionally, anaemia of chronic disease can occur in patients with chronic ALD [10,11]. Conversely, some alcoholics develop iron overload [1,3,12]. It has been documented that chronic alcohol consumption in moderate to excess amounts leads to elevated serum ferritin concentration and transferrin saturation, and can result in increased hepatic iron stores [13]. Additionally, increased intestinal iron absorption has been observed in patients with chronic alcoholic disease [14]. Both iron and ethanol individually cause oxidative stress and lipid peroxidation and the cumulative effects of ethanol and iron on liver cell damage, in patients with ALD, exacerbate liver injury [15–17].

As there is no physiological way of eliminating excess iron, iron homoeostasis is regulated primarily by iron absorption. The process of intestinal non-haeme iron uptake starts with the reduction in ferric iron to the ferrous form by, the brush border enzyme, ferrireductase DCYTB (CYBRD1, duodenal cytochrome b reductase 1) [18]. Iron is then transported across the apical membrane of duodenal enterocytes by means of the divalent metal ion transporter 1 (DMT1, SLC11A2, NRAMP2, DCT1) [19,20]. From the enterocyte, iron is exported across the basolateral membrane by ferroportin (FPN1, SLC40A1, IREG1, MTP1) [21–23]; a ferroxidase hephaestin (HEPH) oxidizes iron to its soluble and non-reactive ferric state, which is then delivered to transferrin [24]. HFE, responsible for hereditary haemochromatosis, is another regulatory protein associated with iron metabolism [25]. HFE affects the interaction of transferrin-bound iron with the transferrin receptor (TFR1, TFRC). Binding of HFE to TFR1 lowers its affinity for iron-transferrin, resulting in a reduction of cellular iron uptake [26,27]. HFE forms complexes also with transferrin receptor 2 (TFR2) [28]. The HFE/TFR2 complex is thought to serve as an iron sensor that regulates hepcidin expression [29]. Hepcidin, a key hormone in the regulation of iron metabolism [30], is produced mainly in the liver. Hepcidin appears to act *via* binding to and internalization of ferroportin and its subsequent degradation [31,32]; although, evidence that hepcidin inhibits apical uptake *via* DMT1 is also available [33]. Hepcidin was found to be up-regulated by iron overload and down-regulated by iron deficiency anaemia and hypoxia [30,34–36]. It has been documented that hepcidin is decreased in patients with haemochromatosis [37–39]. In addition to its response to iron homoeostasis, hepcidin is induced by inflammation [30]. Recently, with the use of animal models, ethanol was shown to down-regulate the expression of hepcidin in the liver which resulted in elevated expression of the iron transporters DMT1 and ferroportin in the duodenum [40,41]. Deregulation of hepcidin synthesis may be one of the causes of iron disturbances during chronic alcohol consumption.

So far, the effect of ethanol on iron uptake *via* duodenal iron transporters and its relation to hepcidin have only been analysed using cell lines and animal models [40–43]. Therefore, the aim of this study was to evaluate the expression of duodenal iron transporters both on mRNA and protein levels and their relation to hepcidin in alcoholic patients either with anaemia, iron overload or normal iron stores.

## Materials and methods

### Patients

A total of 54 individuals (35 male, 19 female), mean age of 57.4 years, ranging from 25 to 82 years were enrolled in the study. The diagnosis of ALD (*N* = 24) was based on patients' history of consumption of more than 30 g alcohol per day, presence of elevated serum AST (aspartate aminotransferase, EC 2.6.1.1) or ALT (alanine aminotransferase, EC 2.6.1.2) and GGT (gamma-glutamyltransferase, EC 2.3.2.2) activity and sonographically observed fatty changes in the liver (liver steatosis). According to laboratory parameters, ALD patients were categorized into three subgroups: ALD with anaemia (*N* = 8), ALD with iron overload (*N* = 6) and ALD without overload (*N* = 10). First, patients were divided according to the presence of anaemia (haemoglobin levels <11 g/dl). These patients had minor decreases in serum iron parameters, however, they did not meet criteria for iron deficiency anaemia (serum ferritin <20 μg/l, haemoglobin <11.0 g/dl and transferrin saturation <16%). Patients without anaemia were then divided according to the presence of iron overload, defined as elevated ferritin levels (cut-off: 200 μg/l for women and 250 μg/l for men) or increased transferrin saturation (cut-off = 45%). The control group (*N* = 30) had an upper GI (gastrointestinal) endoscopy to evaluate their dyspeptic symptoms and their iron parameters were in normal ranges (serum iron 11–26 μmol/l, serum ferritin male 30–250 μg/l, female 30–200 μg/l, transferrin saturation 20–45%). Controls were participants of another study by our group concerning the gene expression in haemochromatosis [44]. To analyse the effect of *HFE* gene mutations, the genotyping for C282Y, H63D and S65C mutations of *HFE* gene was performed using the PCR-RFLP method, as described previously [45]. DNA for *HFE* genotyping was available from 23 ALD patients and from 10 controls. Patients were recruited at our outpatient department between 2005 and 2009. Informed consent was obtained from all patients and the study was approved by the Ethics Committee of the Third Faculty of Medicine, Charles University and conducted in accordance with the Helsinki Convention.

### Sample collection

Duodenal biopsy samples were obtained from 54 individuals during GI endoscopy. For RNA analysis, samples were stored at −20°C in RNAlater solution (Sigma-Aldrich, St. Louis, MO, USA) prior to RNA isolation and for protein analysis at −80°C prior to protein isolation.

### RNA isolation and real-time quantitative polymerase chain reaction

Total RNA was isolated from RNAlater-stored duodenal biopsies by using an RNAeasy MiniKit (Qiagen, Hilden, Germany), and included DNAse digestion according to manufacturer's instructions. After estimation of RNA integrity by using gel electrophoresis and determination of each sample concentration, one sample was found to be inadequate for further analysis. The following analyses were carried out as previously described in detail [44]. Briefly, cDNA synthesis was performed using a reverse transcription kit TaqMan Reverse Transcription Reagents (Applied Biosystems, Foster City, CA, USA) with random primers according to the manufacturer's instructions. Real-time quantitative PCR was performed using an ABI Prism 7000 Sequence Detection System (Applied Biosystems) and commercially available kits: Taq Man Universal PCR Master Mix and Sybr green PCR Master Mix (Applied Biosystems). For the amplification of *FPN1*, *TFR1*, *HFE* and *GAPDH cDNA*, Applied Biosystems pre-designed gene expression assays were used: *FPN1* – Hs00205888_m1, *TFR1* – Hs00174609_m1, *HFE* – Hs00373474_m1 and *GAPDH* – Hs99999905_m1. For amplification of *DMT1*, *DCYTB* and *HEPH* cDNA, previously described analyses were used [46]. *DMT1* analysis was performed on the *DMT1(IRE)* variants because these are the isoforms that are regulated by iron status in cell [47]. All data were normalized to the amount of *GAPDH* cDNA in the sample. The 2^−ΔΔCT^ method was used to calculate relative changes in gene expression.

### Western blot analysis

Because of the limited amount of biological material, Western blot analyses were performed only on duodenal iron transporters and coupled oxido-reductases (DMT1, ferroportin, DCYTB and hephaestin). After protein extraction from duodenal biopsy by using the RIPA buffer (Sigma-Aldrich) and determination of concentration, seven samples were found to be inadequate for further analysis with Western blot. A small amount of tissue, obtained by biopsy, was detected in two samples; therefore, the Western blot analysis could only be performed for hephaestin and DMT1 detection in these samples.

Western blot analyses of DMT1 (IRE variants), DCYTB, ferroportin, hephaestin and actin (loading control) levels were performed using goat polyclonal antibodies NRAMP 2 and Hephaestin against human DMT1 and hephaestin (Santa Cruz Biotechnology, Santa Cruz, CA, USA), goat polyclonal anti-Cytochrome b reductase 1 antibody against human DCYTB (Everest Biotech, Upper Heyford, UK), rabbit polyclonal antibody MTP11-A against human ferroportin (Alpha Diagnostic International, San Antonio, TX, USA) and mouse monoclonal antibody against human actin (Sigma-Aldrich). Analysis was carried out as previously described [44]. Briefly, proteins separated using SDS-PAGE were blotted onto a 0.2 μm nitrocellulose membrane for 2 hrs at 0.25 A, by using a MiniProtean II blotting apparatus (Bio-Rad, Hercules, CA, USA). The membrane for DMT1, ferroportin, DCYTB and hephaestin was blocked with 5% BSA in TBS (100 mM Tris-HCl, 150 mM NaCl, pH = 7.5), whereas, for actin, 5% non-fat milk in TBS was used. The washed membrane was incubated with the relevant primary antibody. After incubation (overnight, 4°C), the washed membrane was incubated for 2 hrs with the corresponding horseradish peroxidase-conjugated secondary antibody, which was then detected by using enhanced chemiluminescence with Supersignal reagent (Pierce, Rockford, IL, USA) and a LAS 1000 CCD device (Fuji; Fujitsu Limited, Tokyo, Japan). Band intensities were quantified by densitometry and the data were analysed by using ImageJ software (version 1.42q; NIH, USA, available on http://rsb.info.nih.gov/ij/).

### ELISA

To evaluate the role of hepcidin, a subgroup of 24 individuals, whose serum was available for hepcidin analysis, was created. It represented samples from eight controls and 16 ALD patients, which were further divided into the following subgroups: ALD with anaemia (*N* = 8), without overload ALD (*N* = 6), and ALD with iron overload (*N* = 2). Mature bioactive hepcidin was measured in serum samples by using a commercial ELISA (EIA-4705) kit (DRG International Inc., Springfield, NJ, USA) according to the manufacturer's instructions. In both patients and controls, blood sample was drawn between 7.30 and 8.30 a.m. after overnight fasting.

### Statistical analysis

Data are expressed as mean ± SEM. Because of non-normality of the measured variables, the non-parametric Mann–Whitney test or the Kruskal–Wallis anova with multiple comparison tests were used as needed. Correlations were assessed using the Spearman rank method. *P* value less than 0.05 was considered significant. Statistical analyses were performed using the Statistica program (Version 9; StatSoft, Tulsa, OK, USA) and the GraphPad Prism program (Version 5.00; GraphPad Software, Inc., San Diego, CA, USA).

## Results

### Clinical and laboratory characteristics of patients

Laboratory parameters of controls and ALD group are shown in Table 1. DNA genotyping showed that among ALD patients, one was found to be a C282Y/H63D compound heterozygote, one was a H63D homozygote, three were H63D heterozygotes and one was a S65C heterozygote. Tested controls were wild-type for all three tested mutations. In further analyses, gene expression values of individuals with *HFE* gene mutations were not outliers or extremes and were within the non-outlier range of values measured in their respective subgroups.

**Table 1:** Clinical characteristics and iron metabolism phenotype in patients and controls

Variable	ALD (*N* = 24)	Controls (*N* = 30)	*P*-value
Age (years)	57.38 ± 1.57	57.40 ± 2.85	0.4431
Gender	17M/7F	18M/12F	0.5673
Serum iron (μmol/l)	18.03 ± 1.94	17.55 ± 0.67	0.6240
Serum ferritin (μg/l)	296.22 ± 96.11	151.0 ± 13.83	0.9640
Transferrin saturation (%)	32.22 ± 4.88	31.22 ± 0.96	0.4455
Hb (g/dl)	12.66 ± 0.44	14.00 ± 0.31	0.0138
Ht (%)	37.16 ± 1.16	41.48 ± 0.93	0.0058
ALT (μkat/l)	0.62 ± 0.05	0.54 ± 0.05	0.0478
AST (μkat/l)	0.81 ± 0.09	0.47 ± 0.03	0.0003

Data are presented as arithmetic mean ± SEM. Hb: haemoglobin; Ht: haematocrit; ALT: alanine aminotransferase; AST: aspartate aminotransferase. Normal ranges: serum iron (11–26 μmol/l), serum ferritin (male 30–250 μg/l, female 30–200 μg/l), transferrin saturation 20–45%, Hb (male 13.0–18.0 g/dl, female 11.5–16.0 g/dl), Ht (male 38–54%, female 35–47%), ALT (0.1–0.75 μkat/l) and AST (0.1–0.75 μkat/l).

### RNA expression

**Fig. 1:**
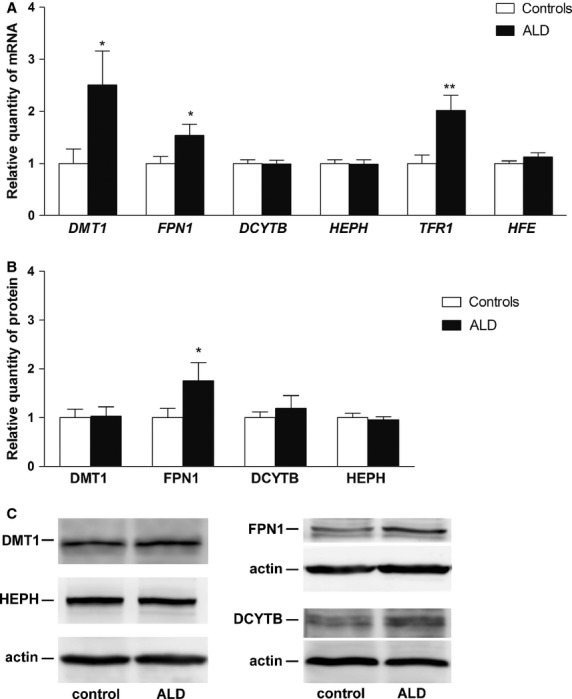
Gene expression of the analysed molecules in controls and patients with alcoholic liver disease (ALD). (**A**) mRNA expression of *DMT1*, *FPN1*, *DCYTB*, *HEPH*, *TFR1* and *HFE*. (**B**) Protein expression of DMT1, FPN1, DCYTB and HEPH. (**C**) Western blot analysis of DMT1, FPN1, DCYTB, HEPH and actin (loading control). Results are depicted as means ± SEM. Statistically significant differences as compared with the control group are indicated by * *P* < 0.05, ** *P* < 0.01 and *** *P* < 0.001.

Gene expression at the mRNA level was measured for *DMT1*, *FPN1*, *DCYTB*, *HEPH*, *HFE* and *TFR1*. A significant increase in the ALD group was found when *DMT1*, *FPN1* and *TFR1* were examined (2.51-fold, *P* = 0.0147, 1.54-fold, *P* = 0.0342, and 2.02-fold, *P* = 0.0011, respectively). Gene expression of the other tested molecules (*DCYTB*, *HEPH* and *HFE*) was not significantly different in ALD patients compared to controls (Fig. 1A).

**Fig. 2:**
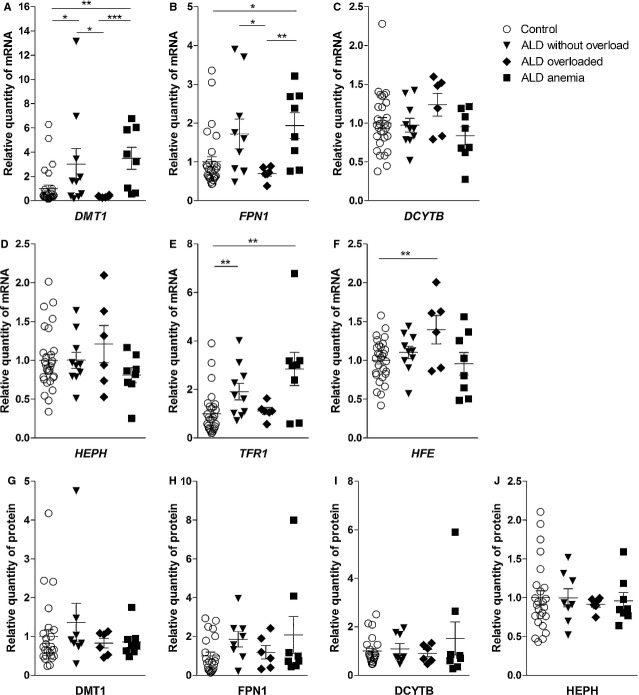
Gene expression of the analysed molecules in controls, patients with alcoholic liver disease without iron overload (ALD without overload), with iron overload (ALD overloaded), and anaemia (ALD anaemia). (**A**) mRNA expression of *DMT1*, (**B**) *FPN1*, (**C**) *DCYTB*, (**D**) *HEPH*, (**E**) *TFR1*, (**F**) *HFE*. (**G**) Protein expression of DMT1, (**H**) FPN1, (**I**) DCYTB, (**J**) HEPH. Results are depicted as means ± SEM. Statistically significant differences are indicated by *P < 0.05, ** *P* < 0.01 and *** *P* < 0.001.

To study the ALD group in more detail, we divided the group into three subgroups: (*i*) patients with the signs of iron overload, (*ii*) patients without iron overload and (*iii*) patients with anaemia according to parameters defined in Materials and Methods. A significant increase in *DMT1* and *FPN1* mRNA was found in the ALD subgroup without iron overload compared to ALD with iron overload (3.02 *versus* 0.34, *P* = 0.0312 and 1.72 *versus* 0.70, *P* = 0.0420, respectively). Moreover, a significant elevation in *DMT1* and *TFR1* mRNA expression was observed in ALD patients without iron overload compared to controls (3.02-fold, *P* = 0.0479, and 1.91-fold, *P* = 0.0066, respectively). Although *FPN1* gene expression was also increased compared to controls, the increase did not reach statistical significance (1.72-fold, *P* = 0.0599). When the ALD subgroup with anaemia was examined, a significant increase in *DMT1*, *FPN1* and *TFR1* mRNA levels was observed compared with controls (3.50-fold, *P* = 0.0018, 1.93-fold, *P* = 0.0115 and 2.84-fold, *P* = 0.0075, respectively). The same applies to comparison of the ALD anaemia subgroup to the ALD iron overload patients when *DMT1* and *FPN1* mRNA were analysed (3.50 *versus* 0.34, *P* = 0.0007 and 1.93 *versus* 0.70, *P* = 0.0080, respectively). Additionally, the *HFE* mRNA level was elevated in ALD patients with iron overload compared with controls (1.40-fold, *P* = 0.0376) (Fig. 2A–F).

### Protein expression

To investigate whether the changes in mRNA expression correlated with changes in protein expression, we examined DMT1, ferroportin, DCYTB and hephaestin levels by using Western blotting.

There was a significant increase in ferroportin protein expression in ALD patients compared with controls (1.76-fold, *P* = 0.0495). Unchanged *DCYTB* and *HEPH* mRNA expression was paralleled by unchanged respective protein expression (1.20-fold, *P* = 0.8292 and 0.96-fold, *P* = 0.8898, respectively). DMT1 protein expression was not different in the ALD group compared with controls (1.03-fold, *P* = 0.3879) (Fig. 1B). Further analysis of the ALD group, divided based on iron overload or anaemia, revealed no significant differences in protein expression. The ferroportin protein expression had the same pattern as seen at the mRNA level; however, the significance found in the ALD group was lost and the increase in ferroportin protein in ALD patients without iron overload and anaemia did not reach statistical significance compared with controls (1.90-fold, *P* = 0.0746 and 2.08-fold, *P* = 0.1685, respectively; Fig. 2G–J).

### Relationships among gene expression of tested molecules

Relationships among DMT1, FPN1, DCYTB, HEPH, TFR1 and HFE expression were analysed by using the Spearman rank correlation. When all individuals regardless of underlying disease were evaluated, there was a positive association among mRNA expression of the tested molecules. The strongest correlation was found between *DMT1* and *FPN1* (*r* = 0.778, *P* < 0.0001), between *DCYTB* and *HEPH* (*r* = 0.687, *P* < 0.0001) and between *DMT1* and *TFR1* (*r* = 0.660, *P* < 0.0001; Table 2).

**Table 2:** Associations among analysed molecules—mRNA level

	mRNA				
	
	*HFE*	*TFR1*	*HEPH*	*DCYTB*	*FPN1*
***DMT1***					
ALD	ns	0.613**	ns	ns	0.863***
Controls	ns	0.634***	ns	ns	0.683***
All	ns	0.660***	ns	ns	0.778***
***FPN1***					
ALD	ns	0.593**	ns	ns	
Controls	ns	0.523**	0.593***	0.364**	
All	ns	0.591***	0.447***	0.322*	
***DCYTB***					
ALD	0.600**	ns	0.570**		
Controls	ns	ns	0.790***		
All	0.468***	ns	0.687***		
***HEPH***					
ALD	ns	ns			
Controls	0.414*	ns			
All	0.399**	0.315*			
***TFR1***					
ALD	ns				
Controls	0.604***				
All	0.452***				

Spearman rank correlation, only statistically significant data are shown, statistically significant differences are indicated by

* *P* < 0.05,

** *P* < 0.01 and

*** *P* < 0.001, ns - not significant.

When the gene expression, at the protein level, in the cohort of all individuals was investigated, a positive relationship between the following proteins was found: DCYTB and ferroportin (*r* = 0.632, *P* < 0.0001), DMT1 and hephaestin (*r* = 0.580, *P* < 0.0001), ferroportin and hephaestin (*r* = 0.482, *P* = 0.0008), DCYTB and hephaestin (*r* = 0.391, *P* = 0.0080) and DMT1 and DCYTB (*r* = 0.314, *P* = 0.0358; Table 3).

**Table 3:** Associations among analysed molecules—protein level

	Proteins			
	
	HEPH	DCYTB	FPN1	Hepcidin
***DMT1***				
ALD	0.792***	0.471*	ns	ns
Controls	0.490*	ns	ns	ns
All	0.580***	0.314*	ns	ns
***Hepcidin***				
ALD	ns	ns	ns	
Controls	ns	ns	ns	
All	ns	ns	ns	
***FPN1***				
ALD	0.428*	0.719***		
Controls	0.580**	0.601**		
All	0.482***	0.632***		
***DCYTB***				
ALD	0.485*			
Controls	ns			
All	0.391**			

Spearman rank correlation, only statistically significant data are shown, statistically significant differences are indicated by

* *P* < 0.05,

** *P* < 0.01 and

*** *P* < 0.001, ns - not significant.

When all individuals were analysed together, a correlation between gene expression at the mRNA level and the protein level was found only for hephaestin (*r* = 0.338, *P* = 0.0215).

When correlations in ALD and controls were investigated separately, positive relationships were found, and are summarized in Tables2 and 3.

### Relationships among gene expression of tested molecules and iron parameters

The association between gene expression of the analysed molecules and serum iron parameters was also tested. When all participants were examined together, a correlation was found between serum ferritin and the mRNA of *DMT1* (*r* = −0.484, *P* = 0.0038) and *FPN1* (*r* = −0.447, *P* = 0.0080). Serum iron correlated at the mRNA level with *FPN1* (*r* = −0.344, *P* = 0.0373). The same applies to the correlation between transferrin saturation and mRNA: *DMT1* (*r* = −0.451, *P* = 0.0108), *FPN1* (*r* = −0.432, *P* = 0.0153) and *TFR1* (*r* = −0.384, *P* = 0.0328). There were no correlations between *DCYTB*, *HEPH* and *HFE* and any of tested iron parameters. When protein expression was examined in the cohort of all participants, no correlation was found between any protein or iron parameter.

With respect to ALD patients analysed separately, there were relationships between serum ferritin and *DMT1* mRNA (*r* = −0.465, *P* = 0.0252) and *FPN1* mRNA (*r* = −0.448, *P* = 0.0318). Transferrin saturation correlated with *DMT1* mRNA (*r* = −0.465, *P* = 0.0289) and serum iron with *TFR1* mRNA (*r* = −0.414, *P* = 0.0495) in ALD patients. Among controls, we found inverse relationship of serum ferritin and *DMT1* mRNA expression (*r* = −0.772, *P* = 0.0053) and ferroportin and DCYTB protein expression (*r* = −0.700, *P* = 0.0358, *r* = −0.766, *P* = 0.0159, respectively). Serum iron correlated with DMT1 protein expression (*r* = −0.641, *P* = 0.0182), and transferrin saturation correlated with DMT1 protein expression (*r* = −0.910, *P* = 0.0017).

Transferrin saturation *versus* serum ferritin and transferrin saturation *versus* serum iron were correlated with each other in all individuals (*r* = 0.747, *r* = 0.863, *P* < 0.0001 for both comparisons listed). The same also applies to correlations between serum iron *versus* serum ferritin (*r* = 0.500, *P* = 0.0031).

### Hepcidin analysis

**Fig. 3:**
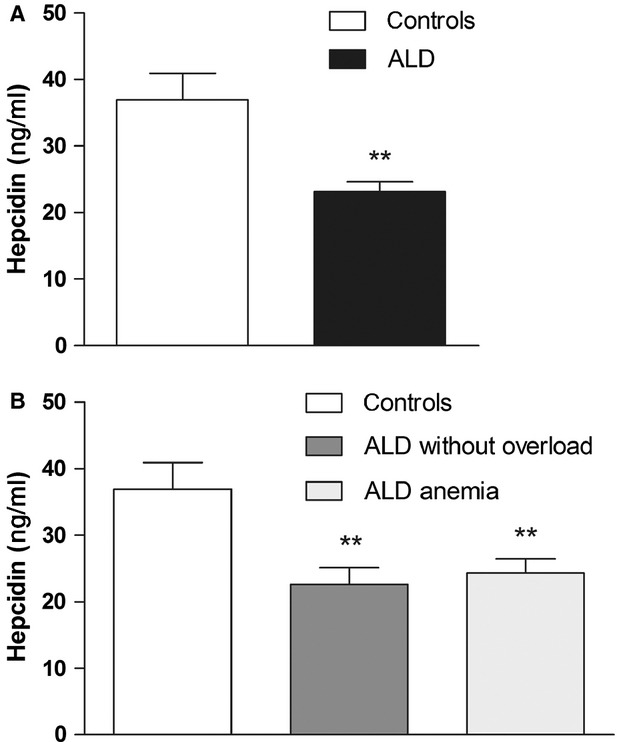
Serum hepcidin levels (**A**) in controls and patients with alcoholic liver disease (ALD) (**B**) in controls, patients with alcoholic liver disease without iron overload (ALD without overload) and anaemia (ALD anaemia). Results are depicted as means ± SEM. Statistically significant differences as compared with the control group are indicated by * *P* < 0.05, ** *P* < 0.01 and *** *P* < 0.001.

To evaluate the regulation of iron metabolism in more detail, the relationship of serum hepcidin and expression of analysed molecules was studied as well. The sera for hepcidin measurements were available from 24 individuals (ALD *N* = 16, controls *N* = 8). A significant decrease in serum hepcidin was observed in ALD patients compared with controls (23.16 ng/ml *versus* 36.93 ng/ml, *P* = 0.0010) (Fig. 3A). When the ALD group was divided based on iron overload or anaemia, significant changes in serum hepcidin levels were found in all ALD subgroups compared with controls: ALD patients without iron overload (22.67 ng/ml *versus* 36.93 ng/ml, *P* = 0.0081) and ALD patients with anaemia (24.30 ng/ml *versus* 36.93 ng/ml, *P* = 0.0027; Fig. 3B). Because of small number of samples in the ALD subgroup with iron overload (*N* = 2), this subgroup was not evaluated, although decreased hepcidin levels were also detected in this group (20.10 ng/ml). Interestingly, when these subgroups were compared to each other, no differences were detected (Fig. 3B). The relationship between serum hepcidin level and gene expression both at the RNA and protein levels was analysed, but no correlation was found. In addition, hepcidin levels did not correlate with any of the measured iron parameters regardless of data analysis techniques or grouping methods, *i.e*. ALD and controls separately or all participants taken together.

## Discussion

In this study, iron metabolism in alcoholic patients with normal iron indices, iron overload and anaemia was examined. Gene expression of molecules participating in iron absorption in duodenum (DMT1, ferroportin, DCYTB and hephaestin) was analysed both at mRNA and protein levels. Additionally, *TFR1* and *HFE* mRNA expression was investigated. Serum hepcidin, a key regulator of iron metabolism, was evaluated as well. This study is, to our knowledge, the first to investigate duodenal gene expression of iron transport molecules both at the mRNA and protein level, together with the serum hepcidin level in ALD patients.

The effect of alcohol consumption on hepcidin expression has been previously demonstrated [40,41]. It has been shown, by using animal models, that alcohol down-regulates hepcidin expression, which affects ferroportin and DMT1 and leads to increased iron absorption in the duodenum. It has been documented that ethanol down-regulates hepcidin promoter activity and DNA binding activity of transcription factor C/EBPα [40,41]. In the present study, hepcidin levels were decreased in ALD patients compared with controls, supporting the abovementioned mechanism of alcohol on hepcidin expression in humans. We found a significant elevation of FPN1 at both the mRNA and protein level and an increase in *DMT1* and *TFR1* mRNA in ALD patients. However, the elevation of *DMT1* mRNA was not paralleled by the elevation of DMT1 protein. It could be speculated that some post-transcriptional mechanisms are involved *e.g*. IRE/IRP system [48], Ndfips/WWP2 system responsible for ubiquitination and degradation of DMT1 [49], or PAP7 which inhibition was shown to cause a reduction in the expression of DMT1 (IRE) protein but not mRNA [50]. The regulation by miRNA can also be considered but the only miRNA documented to affect DMT1 acts on the non-IRE variant [51]. Also, the heterogeneity of patients' genetic background or the small sample size can be the reason of this discrepancy. Further analysis of the ALD subgroups showed that at the mRNA level, there was a significant increase in *DMT1* and *FPN1* in ALD patients without iron overload compared with ALD with iron overload and a significant increase in *DMT1* and *TFR1* mRNA when compared with controls. Additionally, hepcidin levels in these subgroups were significantly lower compared with controls. Thus, it can be hypothesized that in ALD patients, chronic alcohol consumption decreases hepcidin levels, which results in up-regulation of *DMT1* and *FPN1* mRNA in patients with yet normal iron indices. It could be expected that when iron stores become increased, the iron burden is sensed to be inappropriately high by the ‘iron status’ hepcidin regulatory pathway (probably HJV/BMP and/or HFE/TFR2 pathway) and decreased hepcidin levels tend to normalize to compensate iron absorption. This would lead to suppression of increased *DMT1* and *FPN1* mRNA levels in ALD patients with iron overload as seen in our study. However, we did not detect this normalization of hepcidin levels in the ALD iron overload subgroup, probably because of limited number of serum samples; thus we can only speculate at this point. It can be argued that these two factors—alcohol, which tends to decrease hepcidin expression and physiological regulation, which tends to compensate for high iron stores by increasing hepcidin expression, act on various regulatory pathways and this interaction finally establishes some sort of balance; an analogous situation has been documented in treated and untreated haemochromatosis patients [52–54]. This is in agreement with another study where the effect of alcohol together with iron was investigated [42]. In the study, animal models were used and the results showed that iron elevated and alcohol decreased liver hepcidin expression. Alcohol was shown to suppress up-regulation of *hepcidin* mRNA in iron-overloaded rats to levels similar to those in control animals. The duodenal ferroportin expression was elevated in alcohol-treated mice. When both factors were investigated together duodenal ferroportin expression was increased and reached levels between alcohol-treated and iron-treated mice. In addition, the iron-induced increases in the DNA binding activity of C/EBPα were diminished by alcohol to levels found in controls. Similar observations using *HFE* knockout mice were documented in another study [43], which also suggested that alcohol decreases hepcidin expression independently of the HFE pathway, possibly by alcohol-induced hypoxia.

We also investigated ALD patients with anaemia. The pathogenesis of anaemia in ALD is complex: it may include splenic pooling and haemolytic anaemia caused by hypersplenism; chronic bleeding into the gastrointestinal tract resulting in iron deficiency; secondary malnutrition, leading to anaemia with folic acid deficiency; anaemia as a consequence of the direct toxic effect of alcohol on erythrocyte precursors in bone marrow [10]. These symptoms lead to hypoxia, anaemia and iron deficiency, which all inhibit hepcidin synthesis *via* several pathways and corresponding mediators: hypoxia inducible factor α, erythropoietin (EPO), growth differentiation factor 15 and twisted gastrulation protein homologue 1. In iron deficiency, the regulation pathway for iron status includes activity of the BMP/HJV and HFE/TFR2 complex [55,56]. On the other hand, anaemia of chronic disease can occur in patients with chronic ALD [10], when hepcidin expression is induced by inflammatory stimuli (*e.g*. IL-6) [55,56]. Finally, with chronic alcohol consumption, the effect of ethanol on hepcidin promoter activity and the DNA binding activity of transcription factor C/EBPα, must also be considered [41]. All these factors may have played a role in our ALD patients with anaemia and the final level of hepcidin represented the combined effect of all these various pathways. We also found decreased hepcidin level in our ALD patients with anaemia, which was consistent with the abovementioned facts. The effect of increased hepcidin synthesis because of anaemia of chronic disease seems to be minor, especially when the erythroid demand for iron is thought to be a more powerful regulator of hepcidin expression than inflammation [57]. Consequently, these patients displayed increased mRNA expression of *DMT1* and *FPN1* in the duodenum, demonstrating the efforts of the organism to increase iron absorption in response to iron needs associated with enhanced erythropoiesis. However, these elevations were not detected at the protein level of DMT1 and FPN1. Although the expression of FPN1 protein was increased, it did not reach statistical significance. Surprisingly, serum hepcidin levels were similar in ALD patients with anaemia (when the effect of anaemia plus ethanol affects hepcidin synthesis) and without anaemia (when only ethanol is implicated). We were not able to distinguish between the effect of erythropoietic stimulation, iron deficiency and ethanol when analysing the combined effect of all factors playing a role in the pathogenesis of anaemia in our ALD patients. It seems that the effect of various mediators on hepcidin expression pathway is not simply additive, and crosstalk among the different pathways, can be hypothesized, to influence different signal transduction intensities. On the other hand, EPO has been proposed to act directly to repress hepcidin through EPOR-mediated regulation of C/EBPα [58], which is also affected by ethanol. It could be possible that this pathway can suppress hepcidin synthesis, but only to some limit; and once the C/EBPα is influenced by a mediator, others are unable to exert their full influence. However, the small sample size of our associated ALD subgroup did not permit definite conclusions in this respect.

*TFR1* mRNA was increased in ALD patients with anaemia and without iron overload. The increase of *TFR1* in ALD patients without overload cannot be explained by iron deficiency and anaemia as in the ALD anaemia subgroup [59], thus the effect of ethanol needs to be considered. It has been shown that ethanol exposure can increase the expression of TFR1 in hepatocytes [60,61]. The increase in TFR1 expression is partially because of the increased activity of iron regulatory proteins (IRPs) linked to the oxidative stress of ethanol metabolism [60]. It can be speculated that a similar mechanism is implicated in duodenal cells; however, this mechanism has yet to be elucidated. In addition, if ethanol can affect *TFR1* mRNA expression in duodenal cells it may also play a role in the expression of other iron transport molecules.

We detected a strong positive correlation between *DMT1* and *FPN1*; *DCYTB* and *HEPH*; and *DMT1* and *TFR1* at the mRNA level, which suggests a coordinated regulation of these genes. We also investigated the association between serum iron parameters and duodenal iron transporters. Significant inverse correlations between *DMT1* mRNA and serum ferritin, in controls and ALD patients, were detected confirming that body iron stores play a role in the regulation of duodenal expression of this transporter. With regard to controls, our observations are in agreement with several studies [54,62]; however, there were other studies that did not find any correlation at all [52,53].

In conclusion, our results demonstrate that serum hepcidin levels are decreased by alcohol consumption resulting in increased expression of iron transporter ferroportin at the mRNA and protein level and *DMT1* at the mRNA level in duodenum of ALD patients. Detailed analyses revealed that these changes were observed in ALD patients without iron overload and ALD patients with anaemia. The increase in duodenal *TFR1* mRNA expression is a consequence of anaemia and probably also an effect of ethanol on duodenal cells. Positive correlations among *DCYTB*, *HEPH*, *DMT1*, *FPN1* and *TFR1* mRNA indicate coordinated regulation of these genes. Further research is required to elucidate the complex pathogenesis of ALD and the effect of ethanol and oxidative stress derived from its metabolism in duodenal cells deserves further investigation.

## References

[1] Chapman RW, Morgan MY, Laulicht M (1982). Hepatic iron stores and markers of iron overload in alcoholics and patients with idiopathic hemochromatosis. Dig Dis Sci.

[2] Latvala J, Parkkila S, Niemelä O (2004). Excess alcohol consumption is common in patients with cytopenia: studies in blood and bone marrow cells. Alcohol Clin Exp Res.

[3] Whitfield JB, Zhu G, Heath AC (2001). Effects of alcohol consumption on indices of iron stores and of iron stores on alcohol intake markers. Alcohol Clin Exp Res.

[4] Lembke A, Bradley KA, Henderson P (2011). Alcohol screening scores and the risk of new-onset gastrointestinal illness or related hospitalization. J Gen Intern Med.

[5] Le Moine O, Hadengue A, Moreau R (1997). Relationship between portal pressure, esophageal varices, and variceal bleeding on the basis of the stage and cause of cirrhosis. Scand J Gastroenterol.

[6] McCormick PA, Walker S, Benepal R (2007). Hypersplenism is related to age of onset of liver disease. Ir J Med Sci.

[7] Stanley AJ, Robinson I, Forrest EH (1998). Haemodynamic parameters predicting variceal haemorrhage and survival in alcoholic cirrhosis. Q J Med.

[8] Toghill PJ, Green S (1979). Splenic influences on the blood in chronic liver disease. Q J Med.

[9] Maruyama S, Hirayama C, Yamamoto S (2001). Red blood cell status in alcoholic and non-alcoholic liver disease. J Lab Clin Med.

[10] Gonzalez-Casas R, Jones EA, Moreno-Otero R (2009). Spectrum of anemia associated with chronic liver disease. World J Gastroenterol.

[11] Lewis G, Wise MP, Poynton C (2007). A case of persistent anemia and alcohol abuse. Nat Clin Pract Gastroenterol Hepatol.

[12] Gleeson D, Evans S, Bradley M (2006). HFE genotypes in decompensated alcoholic liver disease: phenotypic expression and comparison with heavy drinking and with normal controls. Am J Gastroenterol.

[13] Ioannou GN, Dominitz JA, Weiss NS (2004). The effect of alcohol consumption on the prevalence of iron overload, iron deficiency, and iron deficiency anemia. Gastroenterology.

[14] Duane P, Raja KB, Simpson RJ (1992). Intestinal iron absorption in chronic alcoholics. Alcohol Alcohol.

[15] Sumida Y, Nakashima T, Yoh T (2001). Serum thioredoxin elucidates the significance of serum ferritin as a marker of oxidative stress in chronic liver diseases. Liver.

[16] Winterbourn CC (1995). Toxicity of iron and hydrogen peroxide: the Fenton reaction. Toxicol Lett.

[17] Xu Y, Feng Y, Li H (2012). Ferric citrate CYP2E1-independently promotes alcohol-induced apoptosis in HepG2 cells *via* oxidative/nitrative stress which is attenuated by pretreatment with baicalin. Food Chem Toxicol.

[18] McKie AT, Barrow D, Latunde-Dada GO (2001). An iron-regulated ferric reductase associated with the absorption of dietary iron. Science.

[19] Fleming MD, Trenor CC, Su MA (1997). Microcytic anemia mice have a mutation in Nramp2, a candidate iron transporter. Nat Genet.

[20] Gunshin H, Mackenzie B, Berger UV (1997). Cloning and characterization of a mammalian proton-coupled metal-ion transporter. Nature.

[21] Abboud S, Haile DJ (2000). A novel mammalian iron-regulated protein involved in intracellular iron metabolism. J BiolChem.

[22] Donovan A, Brownlie A, Zhou Y (2000). Positional cloning of zebrafish ferroportin1 identifies a conserved vertebrate iron exporter. Nature.

[23] McKie AT, Marciani P, Rolfs A (2000). A novel duodenal iron-regulated transporter, IREG1, implicated in the basolateral transfer of iron to the circulation. Mol Cell.

[24] Harris ZL, Durley AP, Man TK (1999). Targeted gene disruption reveals an essential rolefor ceruloplasmin in cellular iron efflux. Proc Natl Acad Sci USA.

[25] Feder JN, Gnirke A, Thomas W (1996). A novel MHC class I-like gene is mutated in patients with hereditary haemochromatosis. Nat Genet.

[26] Feder JN, Penny DM, Irrinki A (1998). The hemochromatosis gene product complexes with the transferrin receptor and lowers its affinity for ligand binding. Proc Natl Acad Sci USA.

[27] Parkkila S, Waheed A, Britton RS (1997). Association of the transferrin receptor in human placenta with HFE, the protein defective in hereditary hemochromatosis. Proc Natl Acad Sci USA.

[28] Goswami T, Andrews NC (2006). Hereditary hemochromatosis protein, HFE, interaction with transferrin receptor 2 suggests a molecular mechanism for mammalian iron sensing. J Biol Chem.

[29] Gao J, Chen J, Kramer M (2009). Interaction of the hereditary hemochromatosis protein HFE with transferrin receptor 2 is required for transferrin-induced hepcidin expression. Cell Metab.

[30] Nicolas G, Chauvet C, Viatte L (2002). The gene encoding the iron regulatory peptide hepcidin is regulated by anemia, hypoxia, and inflammation. J Clin Invest.

[31] Nemeth E, Tuttle MS, Powelson J (2004). Hepcidin regulates cellular iron efflux by binding to ferroportin and inducing its internalization. Science.

[32] Nemeth E, Ganz T (2006). Regulation of iron metabolism by hepcidin. Annu Rev Nutr.

[33] Mena NP, Esparza A, Tapia V (2008). Hepcidin inhibits apical iron uptake in intestinal cells. Am J Physiol Gastrointest Liver Physiol.

[34] Frazer DM, Wilkins SJ, Becker EM (2002). Hepcidin expression inversely correlates with the expression of duodenal iron transporters and iron absorption in rats. Gastroenterology.

[35] Nicolas G, Bennoun M, Devaux I (2001). Lack of hepcidin gene expression and severe tissue iron overload in upstream stimulatory factor 2 (USF2) knockout mice. Proc Natl Acad Sci USA.

[36] Pigeon C, Ilyin G, Courselaud B (2001). A new mouse liver-specific gene, encoding a protein homologous to human antimicrobial peptide hepcidin, is overexpressed during iron overload. J Biol Chem.

[37] Bridle KR, Frazer DM, Wilkins SJ (2003). Disrupted hepcidin regulation in HFE-associated haemochromatosis and the liver as a regulator of body iron homoeostasis. Lancet.

[38] Kemna EH, Tjalsma H, Podust VN (2007). Mass spectrometry-based hepcidin measurements in serum and urine: analytical aspects and clinical implications. Clin Chem.

[39] Nemeth E, Roetto A, Garozzo G (2005). Hepcidin is decreased in TFR2 hemochromatosis. Blood.

[40] Bridle K, Cheung TK, Murphy T (2006). Hepcidin is down-regulated in alcoholic liver injury: implications for the pathogenesis of alcoholic liver disease. Alcohol Clin Exp Res.

[41] Harrison-Findik DD, Schafer D, Klein E (2006). Alcohol metabolism-mediated oxidative stress down-regulates hepcidin transcription and leads to increased duodenal iron transporter expression. J Biol Chem.

[42] Harrison-Findik DD, Klein E, Crist C (2007). Iron-mediated regulation of liver hepcidin expression in rats and mice is abolished by alcohol. Hepatology.

[43] Heritage ML, Murphy TL, Bridle KR (2009). Hepcidin regulation in wild-type and Hfe knockout mice in response to alcohol consumption: evidence for an alcohol-induced hypoxic response. Alcohol Clin Exp Res.

[44] Dostalikova-Cimburova M, Kratka K, Balusikova K (2012). Duodenal expression of iron transport molecules in patients with hereditary hemochromatosis or iron deficiency. J Cell Mol Med.

[45] Cimburova M, Putova I, Provaznikova H (2005). S65C and other mutations in the haemochromatosis gene in the Czech population. Folia Biol (Praha).

[46] Kovar J, Neubauerova J, Cimburova M (2006). Stimulation of non-transferrin iron uptake by iron deprivation in K562 cells. Blood Cells Mol Dis.

[47] Hubert N, Hentze MW (2002). Previously uncharacterized isoforms of divalent metal transporter (DMT)-1: implications for regulation and cellular function. Proc Natl Acad Sci USA.

[48] Galy B, Ferring-Appel D, Kaden S (2008). Iron regulatory proteins are essential for intestinal function and control key iron absorption molecules in the duodenum. Cell Metab.

[49] Foot NJ, Dalton HE, Shearwin-Whyatt LM (2008). Regulation of the divalent metal ion transporter DMT1 and iron homeostasis by a ubiquitin-dependent mechanism involving Ndfips and WWP2. Blood.

[50] Okazaki Y, Ma Z, Yeh M (2012). DMT1 (IRE) expression in intestinal and erythroid cells is regulated by peripheral benzodiazepine receptor-associated protein 7. Am J Physiol Gastrointest Liver Physiol.

[51] Andolfo I, De Falco L, Asci R (2010). Regulation of divalent metal transporter 1 (DMT1) non-IRE isoform by the microRNA Let-7d in erythroid cells. Haematologica.

[52] Kelleher T, Ryan E, Barrett S (2004). Increased DMT1 but not IREG1 or HFE mRNA following iron depletion therapy in hereditary haemochromatosis. Gut.

[53] Nelson JE, Mugford VR, Kilcourse E (2010). Relationship between gene expression of duodenal iron transporters and iron stores in hemochromatosis subjects. Am J Physiol Gastrointest Liver Physiol.

[54] Stuart KA, Anderson GJ, Frazer DM (2003). Duodenal expression of iron transport molecules in untreated haemochromatosis subjects. Gut.

[55] Pietrangelo A (2011). Hepcidin in human iron disorders: therapeutic implications. J Hepatol.

[56] Viatte L, Vaulont S (2009). Hepcidin, the iron watcher. Biochimie.

[57] Theurl I, Aigner E, Theurl M (2009). Regulation of iron homeostasis in anemia of chronic disease and iron deficiency anemia: diagnostic and therapeutic implications. Blood.

[58] Pinto JP, Ribeiro S, Pontes H (2008). Erythropoietin mediates hepcidin expression in hepatocytes through EPOR signaling and regulation of C/EBPalpha. Blood.

[59] Pietrangelo A, Rocchi E, Casalgrandi G (1992). Regulation of transferrin, transferrin receptor, and ferritin genes in human duodenum. Gastroenterology.

[60] Kohgo Y, Ohtake T, Ikuta K (2005). Iron accumulation in alcoholic liver diseases. Alcohol Clin Exp Res.

[61] Suzuki Y, Saito H, Suzuki M (2002). Up-regulation of transferrin receptor expression in hepatocytes by habitual alcohol drinking is implicated in hepatic iron overload in alcoholic liver disease. Alcohol Clin Exp Res.

[62] Stuart KA, Anderson GJ, Frazer DM (2004). Increased duodenal expression of divalent metal transporter 1 and iron-regulated gene 1 in cirrhosis. Hepatology.

